# Proinsulin-mediated induction of type 1 diabetes in HLA-DR4-transgenic mice

**DOI:** 10.1038/s41598-018-32546-4

**Published:** 2018-09-20

**Authors:** Johan Verhagen, Emma L. Smith, Emily M. Whettlock, Benedict Macintyre, Mark Peakman

**Affiliations:** 1 School of Immunology and Microbial Sciences, Faculty of Life Sciences & Medicine, King’s College London, Guy’s Hospital, London, United Kingdom; 2 UCB Pharma Ltd, Slough, United Kingdom; 3 Present Address: Oncogenes and Tumour Metabolism Laboratory, The Francis Crick Institute, London, UK; 4 Institute of Diabetes, Endocrinology and Obesity, King’s Health Partners, London, UK

## Abstract

Antigen-specific immunotherapy of autoimmune disease currently remains the only potentially curative approach. However, translation of promising pre-clinical results into successful clinical application has proven challenging. In part, this is because pre-clinical findings in mouse models have to be redesigned for human application due to differences in MHC II. To reduce the gap between pre-clinical and clinical studies, we have created a novel mouse model that expresses human HLA-DR4, but no endogenous MHC on antigen-presenting cells. Moreover, human B7.1 (CD80) is expressed in the pancreatic islets under the control of the rat insulin promoter. Although this model does not develop diabetes spontaneously, it is susceptible to the induction of type 1 diabetes by challenging mice with overlapping peptides derived from murine proinsulin-2 in adjuvant. Unlike the NOD model of spontaneous type 1 diabetes, but akin to the human condition, this model does not have a gender bias. Furthermore, similar to the human condition, the disease is characterised by a diverse leucocyte infiltration of the pancreatic islets and the formation of anti-proinsulin auto-antibodies. The model that we report here offers detailed insights into type-1 diabetes and is expected to prove instrumental when studying the mechanism of action in translational, antigen-specific immunotherapy.

## Introduction

Antigen-specific immunotherapy (ASI) represents a promising approach for the directed abrogation of autoimmune diseases, including type 1 diabetes. Although significant advances have been made in recent years to develop peptide antigens suitable for immunotherapy, translation of findings in animal models to the clinic has proven challenging. The non-obese diabetic (NOD) mouse model has been instrumental in addressing many aspects of type 1 diabetes, yet it is not well suited to investigating the efficacy and mechanism of ASI. This is true particularly for peptide-based ASI strategies, in which peptides designed for clinical application are restricted by human MHC molecules. Previous attempts to address this issue, for example by generating NOD mice that express human HLA-DRB1*0401 (HLA-DR4) transgenically instead of endogenous murine MHC class II, have shown limited success, in this case because the human transgene prevented disease development^1^. This problem has, at least in part, been overcome in other models, in which mice are transgenic for HLA-DR4 and ectopically express the co-stimulatory molecule CD80 on pancreatic beta cells under the control of the rat insulin promoter (DR4xRIP-B7.1) to augment local immune responses^2,3^. These animals demonstrate spontaneous diabetes, albeit only at a very advanced age (mean onset of 37 weeks^3^) and with limited penetrance. Concurrently, in our hands, a similar DR4xRIP-B7.1 model did not show any spontaneous disease before the age of 30 weeks. Such low rates of spontaneous disease make it difficult to test therapeutic strategies because of the large numbers needed to reach adequate statistical power, the length of the experiments and the inability to synchronise disease onset.

In an attempt to address these challenges, we have refined these models using an *in vivo*, adjuvanted antigen priming approach that exploits proinsulin as a major autoantigen in the early stages of type 1 diabetes in both humans and mice^4,5^. Mice (C57BL/6 background) expressing a chimaeric MHC containing the human HLA-DR4 peptide-binding region, but deficient in endogenous MHC II, were crossed with mice expressing human B7.1 (CD80) under the control of the proinsulin-2 promoter. We demonstrate here that challenging DR4xRIP-B7.1 mice with overlapping peptides derived from murine proinsulin-2 in adjuvant induces type 1 diabetes in the majority of animals within 20 weeks post-prime and that this disease reflects the human condition.

## Materials and Methods

### Animals

HLA-DR4-transgenic mice (Taconic, Germantown, MD, USA; model 4149) were crossed with RIP-B7.1-transgenic animals (EMMA, Orleans, France; ID 00216) in order to obtain HLA-DR4^+^I-A/IE^−/−^RIP.B7.1^+^ mice. All animals were kept under specific-pathogen-free conditions, in individually ventilated cages, at the KCL Biological Services Unit on 12-hour light/dark cycles with food and water provided ad libitum. Experiments were conducted in accordance with UK Home Office regulations under project licence numbers 707520 and P25D0FA67 held by M. Peakman. All work was subject to assessment and approved locally by Guy’s animal welfare and ethical review board (AWERB).

### Priming antigens

All proinsulin-2 peptides were custom manufactured as acetate salts by Almac (Edinburgh, UK) at >95% purity. The 377-amino acid c-terminal fragment of IA-2 was produced by ProteoGenix (Schiltigheim, France).

### Induction and monitoring of type 1 diabetes

**Figure 1:**
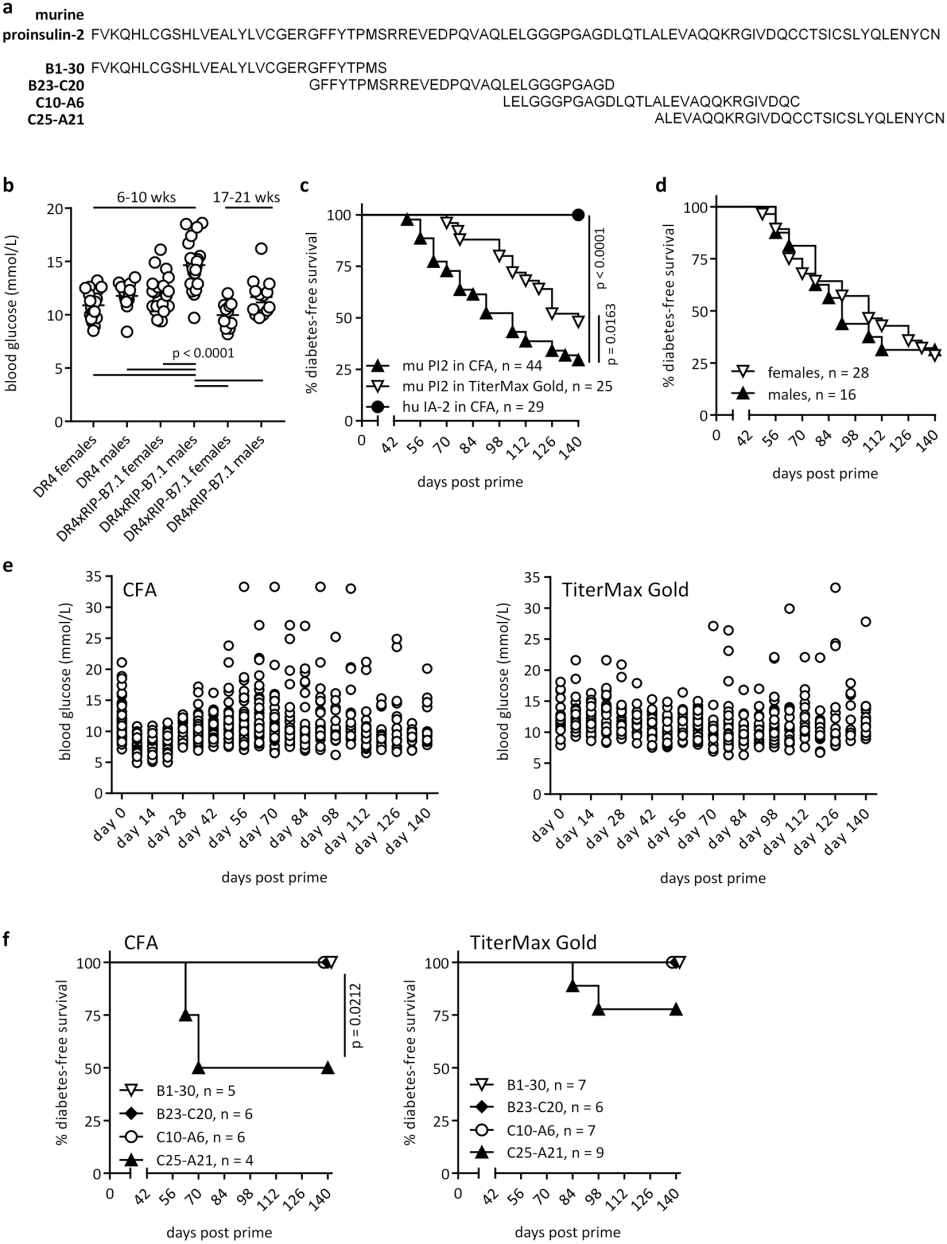
Inducible type 1 diabetes in DR4xRIP-B7.1 mice. (**a**) Overlapping 30-mer peptides derived from murine proinsulin-2, used to induce disease. (**b**) Blood glucose levels of untreated HLA-DR4 (22 ♀, 14 ♂) and DR4xRIP-B7.1 (23 ♀, 23 ♂) aged 6–10 weeks as well as DR4xRIP-B7.1 (11 ♀, 13 ♂) aged 17–21 weeks. Tukey’s multiple comparison test. (**c**) Diabetes incidence in DR4xRIP-B7.1 mice after either one challenge with 100 μg murine proinsulin-2 peptide or 100 μg of IA-2 fragment in CFA s.c. (+200 ng pertussis toxin i.p. on days 0 and 2) or 2 doses of proinsulin-2 peptide in TiterMax Gold on days 0 and 35. Data combined from 4 or more individual experiments. Statistical analysis with Gehan-Breslow Wilcoxon test. (**d**) Diabetes incidence in male versus female mice after challenge with antigen in CFA as in c. (**e**) Blood glucose levels after challenge as in c. CFA, n = 40, TiterMax Gold, n = 25. (**f**) Diabetes incidence in DR4xRIP-B7.1 mice after either one challenge with 100 μg of each individual murine proinsulin-2 peptide in CFA s.c. (+200 ng pertussis toxin i.p. on days 0 and 2) or 2 doses of each proinsulin-2 peptide in TiterMax Gold on days 0 and 14. Statistical analysis with Gehan-Breslow Wilcoxon test.

Mice (aged 6–10 weeks) primed with 100 μg of an equal mixture of overlapping peptides spanning the length of proinsulin-2 (Fig. 1a) in Complete Freund’s Adjuvant (CFA) supplemented with 8 mg/ml Mycobacterium Tuberculosis (Both BD Biosciences, Sparks, MD, USA) s.c. at the base of the tail received 200 ng Pertussis toxin (Sigma, Poole, UK) in PBS i.p. on days 0 and 2. Mice primed with antigen in TiterMax Gold (TiterMax, Norcross, GA, USA) s.c. at the base of the tail received a second dose s.c. distributed over the inguinal region on day 14 or 35. Mice were monitored weekly for hyperglycaemia by a minimal puncture of the tail vein at alternate sides of the tail and analysis using a OneTouch Verio meter (Lifescan, High Wycombe, UK) and for glycosuria using Diastix strips (Bayer, Basel, Switzerland). Mice were considered diabetic following a blood glucose reading >16.7 mmol/l in addition to confirmed glycosuria. Immediately upon detection of diabetes, the project licence dictated that the mice were sacrificed humanely.

### Histology

Pancreata embedded in OCT compound (Cellpath, Newtown, UK) were frozen in isopentane (Sigma) cooled with liquid nitrogen. 8–10 μm sections were fixed in acetone before staining with biotinylated antibodies to CD4, CD8, CD11b, CD11c, GR-1 and B220 (all from eBioscience, Altrincham, UK). Staining was detected using ABC reagent and DAB solution from Vector Labs (Peterborough, UK). Images acquired on a Zeiss Axiovert A1 microscope using Zen software. No cropping or alterations of the images other than changing contrast was used.

### Autoantibody ELISA

Proinsulin-2 peptides were coated onto Maxisorp plates (Nunc, Roskilde, Denmark) in ELISA coating buffer (eBioscience). Diluted sera (in PBS 5% BSA, Sigma) were incubated for 2 hours at room temperature prior to detection with biotin-anti-mouse IgG, Streptavidin-HRP and TMB solution (all from eBioscience) and read at 450 nm.

### Cytokine detection

Splenocytes from DR4xRIP-B7.1 mice challenged with proinsulin-2 peptides in CFA, as above, were restimulated *in vitro* at 1.5 × 10^6^ cells per well in duplicates in round-bottom 96-well plates for 48 hours. Cytokine levels in the cell culture supernatant was determined using a custom Legendplex assay (Biolegend, London, UK) as per the manufacturer’s instructions and analysed on a BD FACScanto II cytometer.

## Results

To promote disease development in HLA-DR4-transgenic mice, animals aged 6–10 weeks were challenged with 30-mer, overlapping peptides that together span the length of murine proinsulin-2 (Fig. 1a), in adjuvant, subcutaneously. Proinsulin-2 rather than proinsulin-1 was chosen due to its greater similarity in sequence and expression pattern to human proinsulin. Although young DR4xRIP-B7.1 mice (6–10 weeks of age), males in particular, exhibit elevated blood glucose levels without manipulation as a result of the RIP-B7.1 transgene, this did not continue into later life (Fig. 1b). To ensure that the transient hyperglycaemia detected occasionally in this model was not mistaken for diabetes, the disease in this model was defined by the very strict criteria of glycosuria in addition to blood glucose levels of >16.7 mmol/L, a diagnosis from which we have not found mice to be able to recover. Judged by these criteria, DR4xRIP-B7.1 mice did not exhibit spontaneous onset of diabetes during follow-up to age 30 weeks (n = 17, 9 ♀, 8 ♂), nor was there any sign of anything but a very low level insulitis even at this advanced age. An adjuvanted approach was therefore required. Because of the reported inhibitory effect of complete Freund’s adjuvant (CFA) on diabetes development, and the potential role of mycobacteria in this process^6,7^, mice were given the immunising peptides in either CFA or TiterMax Gold adjuvant (which contains no mycobacteria). HLA-DR4-transgenic mice that did not co-express RIP-B7.1 did not develop type 1 diabetes with these methods, as also found previously when challenging mice with human proinsulin in adjuvant^8^.

As demonstrated in Fig. 1c, either a single challenge with 100 μg of an equal mixture of the four priming peptides in CFA (with Pertussis toxin given i.p. on days 0 and 2), or two challenges in TiterMax (days 0 and 35), were sufficient to induce type 1 diabetes in a high proportion of animals (approximately 70% and 50%, respectively). Importantly, there was no disease-associated gender bias, with comparable rates of onset in males and females (Fig. 1d). Although the presence of CFA by no means limited disease development, the co-administration of Pertussis toxin led to a temporary reduction in mean blood glucose levels in the first few weeks post-challenge with antigen in adjuvant (days 7–21; Fig. 1e), most likely due to its effect on insulin production^9^. Moreover, the disease induced was clearly mediated by the proinsulin-2 derived peptides and did not result merely from a priming effect of the adjuvant and/or pertussis toxin, as mice treated with the 377-amino acid c-terminal region of human Islet cell Antigen-2 (IA-2) in CFA s.c. and Pertussis toxin i.p. remained diabetes-free throughout the 20-week experiment (Fig. 1c). Finally, we examined which of the four proinsulin-2 derived peptides represented the most diabetogenic region on this HLA-DR4-transgenic background. For this, mice were challenged with 100 μg of each individual peptide in either CFA (with Pertussis toxin administered i.p. on days 0 and 2) or in TiterMax Gold adjuvant (2 doses on day 0 and 14). For both adjuvants, the C25-A21 peptide was the only peptide able to induce diabetes alone (Fig. 1f), albeit less efficiently than when all four peptides were combined.

**Figure 2:**
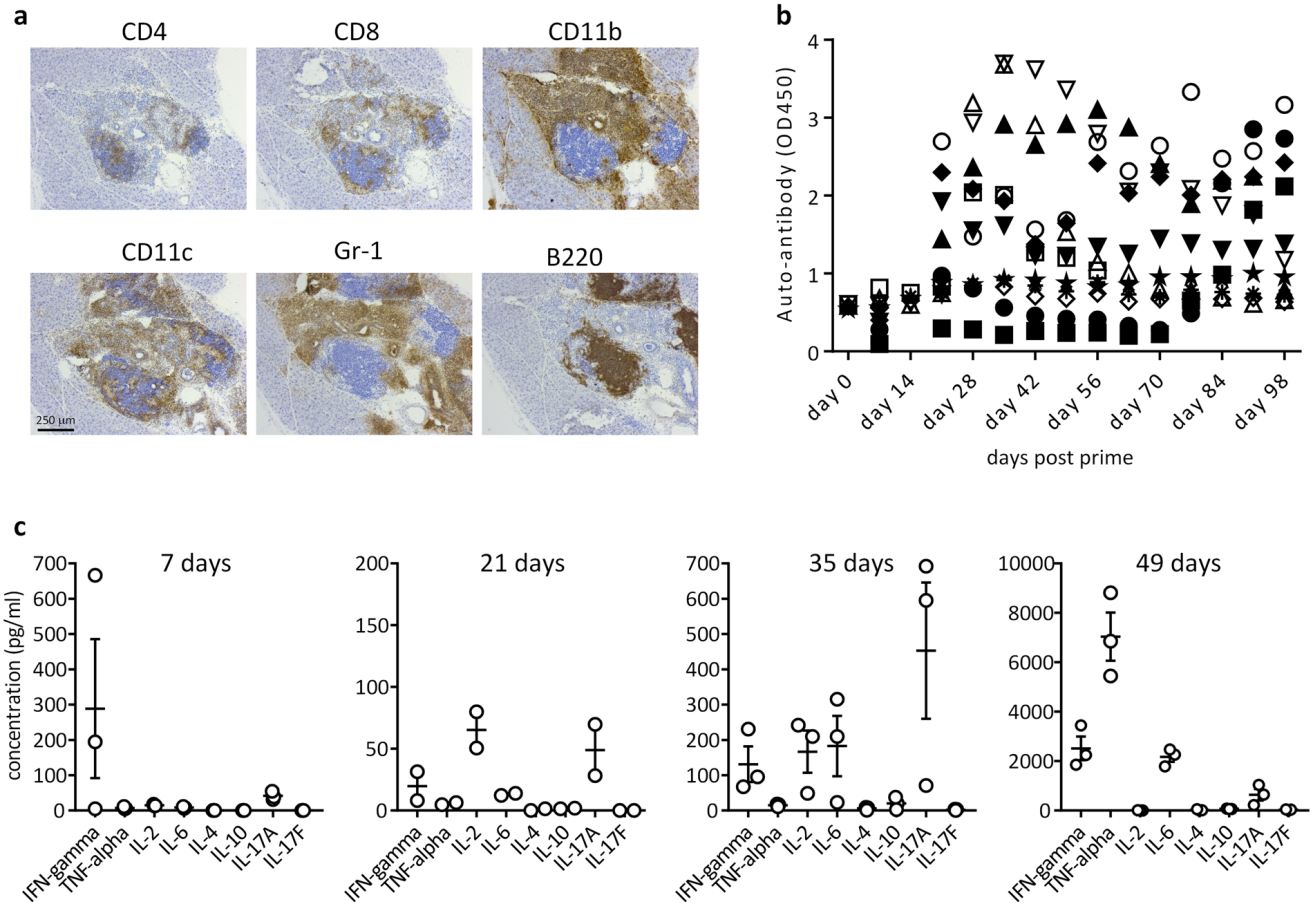
Immunological aspects of inducible type 1 diabetes. (**a**) Representative images of immunohistochemical staining of frozen pancreas sections of DR4xRIP-B7.1 mice with confirmed type 1 diabetes (n > 8 for each staining). 10x objective. (**b**) Detection of serum IgG autoantibody specific for priming peptides as in a. n = 12, combined from 2 separate experiments. Each symbol refers to the same mouse at each time point. (**c**) Cytokine levels in culture supernatant determined by a multiplex bead-based flowcytometric assay. DR4xRIP-B7.1 mice were challenged with 100 μg murine proinsulin-2 peptide in CFA s.c. At 1, 3, 5, or 7 weeks post prime, spleens were removed and splenocytes restimulated *in vitro* with 10 μg/ml of the priming peptides for 48 hrs before sampling supernatant. n = 3 per group, except week 3 (n = 2). Each dot represents the mean of duplicate samples from one individual. Horizontal lines indicate mean values for all individuals. Error bars indicate S.E.M.

In addition to obtaining a diabetic phenotype in this model, we examined its immunological features. Diabetic mice demonstrated a high level of pancreatic islet infiltration (Fig. 2a). Infiltrating cells importantly include CD4^+^ and CD8^+^ T cells but also B lymphocytes and a plethora of myeloid cells. This diverse infiltrate is reminiscent of that seen in the human condition^10^. Moreover, we detected IgG autoantibodies against the priming peptides in the majority of mice from approximately 3 weeks post prime with antigen in adjuvant (Fig. 2b), although seropositivity was not a predictor of disease development. The antibodies generated may thus not play a role in the pathogenesis observed but rather develop purely as a result of the priming response, although this requires further investigation. We were unsuccessful in generating recombinant murine proinsulin-2 to determine reactivity of the antibodies with whole protein, but did establish that they did not recognise human proinsulin (not shown). Finally, the cytokine profile of splenocytes restimulated *in vitro* at 2-week intervals from day 7 post challenge with proinsulin-2 peptides in CFA was examined in order to get an insight into the mechanism of disease development. As demonstrated in Fig. 2c, the immune response to the proinsulin-2 peptides escalated from a mild Th1/Th17 response 1 week post prime to an abundant production of IFN-γ, IL-6 and TNF-α in particular by week 7, the time of earliest diabetes onset. This corroborates the importance of not only T cells, but also myeloid cells in the pathology.

## Discussion

The novel, inducible model we describe here may expedite translation of antigen-based therapies and may prove particularly useful in settings in which there is a requirement to accommodate HLA restriction. This allows for the use of human antigen and potentially human cells to be administered. Moreover, there is no gender bias, unlike the NOD model and spontaneous diabetes in other DR4xRIP-B7.1 models^3^, but akin to the human disease. Because disease is initiated by a known and relevant antigen, proinsulin, this facilitates easier analysis of antigen-specific tolerance induction than in models of spontaneous diabetes, where disease may be mediated by a plethora of antigens. However, one should keep in mind that even though the disease is initiated by a single antigen, this does not necessarily imply that epitope spreading to other islet antigens does not occur.

Although our model expresses a transgenic, human MHC II, it does not have a fully human immune cell repertoire, unlike for example a recently published model using human haematopoietic stem cell-engrafted NSG-HLA-DQ8 transgenic mice^11^. The downside of the latter model is that it does not develop diabetes. This issue was more recently overcome by Tan *et al*.^12^, by creating a HLA-DQ8-transgenic immunodeficient model and transferring in TCR-transgenic, human T cells specific for InsB:9-23 peptide. Still, disease in this model depends on further immunisation with InsB:9-23 in adjuvant and the use of streptozotocin. Although disease in this model is completely mediated by cells of human origin, its use for developing immunotherapy is limited as disease can only be mediated by one epitope and is highly skewed towards the function of one population of TCR-transgenic T cells. Therefore, although our model does not contain cells of human origin, the diversity of the immune response that mediates disease and potentially tolerance may arguably be more representative of what occurs in a typical clinical setting. Both approaches may offer insights that could complement each other in order to optimise ASI.

By inducing disease with each of the four overlapping proinsulin-2 peptides individually, we could demonstrate that the latter end, C25-A21, contained the immunodominant region for diabetes induction in our model. Although care should be taken when comparing spontaneous and induced disease, it would seem that disease in individuals expressing HLA-DR4 is distinct from that in individuals expressing HLA-DQ8 (or the similar I-A^g7^ in mice), where the B9-23 region was previously found to be the dominant diabetogenic peptide^13^.

In our model, neither the presence of the HLA-DR4 transgene nor the use of CFA prohibited the development of diabetes, despite their disease-inhibiting effects in other settings^1,2,6,7^. In fact, CFA induced disease with greater efficacy than TiterMax Gold adjuvant, although the latter may be preferable in some cases as it does not have the predominant adverse effect of the former, i.e. skin lesions at the site of injection.

All together, we suggest that this new model could be instrumental in the future development of novel immunotherapy strategies, be they tolerance induction through the administration of antigen, either alone or combined with carrier vehicles or other immune regulatory agents, or immune cells. The ability to reliably and thoroughly study new treatments in a relevant *in vivo* model should lead to greater safety and efficacy of clinically applied immunotherapy.
